# Compartmentalized profiling of amniotic fluid cytokines in women with preterm labor

**DOI:** 10.1371/journal.pone.0227881

**Published:** 2020-01-16

**Authors:** Gaurav Bhatti, Roberto Romero, Gregory Edward Rice, Wendy Fitzgerald, Percy Pacora, Nardhy Gomez-Lopez, Mahendra Kavdia, Adi L. Tarca, Leonid Margolis

**Affiliations:** 1 Perinatology Research Branch, Division of Obstetrics and Maternal-Fetal Medicine, Division of Intramural Research, *Eunice Kennedy Shriver* National Institute of Child Health and Human Development, National Institutes of Health, U.S. Department of Health and Human Services, Bethesda, Maryland, and Detroit Michigan, United States of America; 2 Department of Biomedical Engineering, Wayne State University College of Engineering, Detroit, Michigan, United States of America; 3 Department of Obstetrics and Gynecology, University of Michigan, Ann Arbor, Michigan, United States of America; 4 Department of Epidemiology and Biostatistics, Michigan State University, East Lansing, Michigan, United States of America; 5 Center for Molecular Medicine and Genetics, Wayne State University, Detroit, Michigan, United States of America; 6 Detroit Medical Center, Detroit, Michigan, United States of America; 7 Department of Obstetrics and Gynecology, Florida International University, Miami, Florida, United States of America; 8 Centre for Clinical Research, University of Queensland, Herston, Queensland, Australia; 9 Section on Intercellular Interactions, *Eunice Kennedy Shriver* National Institute of Child Health and Human Development, National Institutes of Health, U.S. Department of Health and Human Services, Bethesda, Maryland, United States of America; 10 Department of Obstetrics and Gynecology, Wayne State University School of Medicine, Detroit, Michigan, United States of America; 11 Department of Biochemistry, Microbiology, and Immunology, Wayne State University School of Medicine, Detroit, Michigan, United States of America; 12 Department of Computer Science, Wayne State University College of Engineering, Detroit, Michigan, United States of America; Azienda Ospedaliero Universitaria Ospedali Riuniti di Ancona Umberto I G M Lancisi G Salesi, ITALY

## Abstract

**Objective:**

Amniotic fluid cytokines have been implicated in the mechanisms of preterm labor and birth. Cytokines can be packaged within or on the surface of extracellular vesicles. The main aim of this study was to test whether the protein abundance internal to and on the surface of extracellular vesicles changes in the presence of sterile intra-amniotic inflammation and proven intra-amniotic infection in women with preterm labor as compared to the women with preterm labor without either intra-amniotic inflammation or proven intra-amniotic infection.

**Study design:**

Women who had an episode of preterm labor and underwent an amniocentesis for the diagnosis of intra-amniotic infection or intra-amniotic inflammation were classified into three groups: 1) preterm labor without either intra-amniotic inflammation or proven intra-amniotic infection, 2) preterm labor with sterile intra-amniotic inflammation, and 3) preterm labor with intra-amniotic infection. The concentrations of 38 proteins were determined on the extracellular vesicle surface, within the vesicles, and in the soluble fraction of amniotic fluid.

**Results:**

1) Intra-amniotic inflammation, regardless of detected microbes, was associated with an increased abundance of amniotic fluid cytokines on the extracellular vesicle surface, within vesicles, and in the soluble fraction. These changes were most prominent in women with proven intra-amniotic infection. 2) Cytokine changes on the surface of extracellular vesicles were correlated with those determined in the soluble fraction; yet the magnitude of the increase was significantly different between these compartments. 3) The performance of prediction models of early preterm delivery based on measurements on the extracellular vesicle surface was equivalent to those based on the soluble fraction.

**Conclusions:**

Differential packaging of amniotic fluid cytokines in extracellular vesicles during preterm labor with sterile intra-amniotic inflammation or proven intra-amniotic infection is reported herein for the first time. The current study provides insights into the biology of the intra-amniotic fluid ad may aid in the development of biomarkers for obstetrical disease.

## Introduction

Preterm birth (spontaneous and iatrogenic) is the leading cause of perinatal morbidity and mortality [1–6]. The keystone to improving health outcomes in women at risk of preterm birth is a thorough understanding of pathologic processes involved, identification of biomarkers, and implementation of therapeutic interventions. Of the risk factors identified for preterm birth, strong evidence supports the activation of intrauterine inflammatory pathways [7–17]. Consistent with these data, intra-amniotic inflammation due to microbial invasion of the amniotic cavity is an important cause of spontaneous preterm delivery [18–20], and the molecular mechanisms that may be responsible for parturition in this scenario have been extensively studied [18–35].

Proteins present in amniotic fluid, in particular cytokines, are key regulators of parturition, and labor-associated changes in their concentrations, with or without infection at both term and preterm, have been well characterized [36–62]. Until recently, regulatory activity of these proteins was considered to be mediated via soluble autocrine [63–66], paracrine [63, 65, 67], and endocrine [68–70] signaling pathways, by direct engagement with cell-surface receptors. However, it is now established that such mediators are also associated with extracellular vesicles (both ectosomes and exosomes) and are present both on the surface and within the lumen of vesicles [71–74]. Extracellular vesicle-associated proteins, therefore, represent an additional, and as yet uncharacterized, pathway that may contribute to the initiation of labor and delivery at both term and preterm.

Extracellular vesicles have been identified in amniotic fluid [75–87] and available data indicate that exosome concentrations may increase in labor, both at term and preterm [86]. Amnion epithelial and stem cells release extracellular vesicles *in vitro* [83, 84, 88–90] and, therefore, may contribute to the amount of extracellular vesicles in amniotic fluid *in vivo*. Additional sources of extracellular vesicles in amniotic fluid may include the fetal skin, urine, and lung.

Labor and delivery are associated with significant changes in the protein complement of amniotic fluid extracellular vesicles [86]. There is a paucity of data, however, about the role of amniotic fluid extracellular vesicle-associated proteins in preterm labor and delivery and whether their presentation on extracellular vesicles changes in association with intra-uterine inflammation (with or without proven infection).

Herein, we tested the hypothesis that preterm labor with sterile intra-amniotic inflammation and preterm labor with intra-amniotic infection are associated with an increased expression of cytokines in extracellular vesicles present in amniotic fluid, and that such changes will differ between the different extracellular vesicle compartments (internal, surface) and the soluble fraction.

## Materials and methods

### Clinical cohort and study design

A retrospective, cross-sectional study was conducted that included women who had an episode of preterm labor and underwent an amniocentesis for the diagnosis of intra-amniotic infection or intra-amniotic inflammation. Amniotic fluid that was not used for clinical tests was retained for research purposes. These amniotic fluid samples were stored in the Bio-Repository of Wayne State University and the Detroit Medical Center. The Bio-Repository and associated patient data were curated using a commercially available sample inventory and resource management system (BSI Systems, Calverton, MD, USA) at the Perinatology Research Branch of the *Eunice Kennedy Shriver* National Institute of Child Health and Human Development (NICHD), National Institutes of Health, U.S. Department of Health and Human Services (Detroit, MI, USA). The inclusion criteria for this study required an episode of preterm labor, a singleton gestation, a transabdominal amniocentesis performed between 17 and 36 weeks of gestation with microbiologic studies, and a live-born fetus with available data regarding neonatal outcomes. Patients with placenta previa were excluded from the study or if their fetus had a chromosomal abnormality or structural anomaly.

The Institutional Review Boards of Wayne State University and/or National Institute of Child Health and Human Development (NICHD) / National Institutes of Health / U.S. Department of Health and Human Services (Detroit, MI, USA) approved the study. Specimens were collected under the protocols noted herein: Establishment of a Clinical Perinatal Database and a Bank of Biological Materials [WSU IRB# 082403MP2F (5R) and NICHD/NIH# OH98-CH-N001]; Biological Markers of Disease in the Prediction of Preterm Delivery, Preeclampsia and Intra-Uterine Growth Restriction: A Longitudinal Study (WSU IRB#110605MP2F and NICHD/NIH# OH97-CH-N067); Microarray Expression Profiling to Identify Stereoptypic mRNA Profiles in Human Parturition (WSU IRB# 103108MP2F and NICHD/NIH # OH99-CH-N056); Cerebral Palsy: Clinical, Biochemical, Histological, and Biophysical Parameters in the Prediction of Cerebral Palsy in Patients with Preterm Labor and Premature Rupture of Membranes (NICHD/NIH# OH97-CH-N066); The Role of Feto-Maternal Inflammation as a Mechanism of Disease in the Great Obstetric Syndromes (WSU IRB# 075299M1E).

### Clinical definitions

Gestational age was determined by the date of the last menstrual period and confirmed by the first ultrasound examination or by ultrasound examination alone if the sonographic determination of gestational age was inconsistent with menstrual dating. **Term labor** was defined as the presence of regular uterine contractions with a frequency of at least 1 every 10 minutes and cervical changes occurring after 37 weeks of gestation [91]. **Preterm birth** was defined as delivery between 20 and 36^6/7^ weeks of gestation. **Early preterm birth** was defined as delivery between 20 and 31^6/7^ weeks of gestation. **Moderate to late preterm birth** was defined as delivery between 32 and 36^6/7^ weeks of gestation. **Term delivery** was defined as birth ≥ 37 weeks of gestation. **Intra-amniotic inflammation** was diagnosed when the concentration of interleukin 6 (IL-6) in the amniotic fluid was ≥ 2.6 ng/ml [92, 93]. **Microbial invasion of the amniotic cavity** was detected from a positive amniotic fluid culture and/or polymerase chain reaction/electrospray ionization-mass spectrometry (PCR-ESI/MS) result [18, 19, 94–101]. Based on the results of the amniotic fluid culture, PCR-ESI/MS, and amniotic fluid IL-6 concentration, patients were classified into the following groups:

1. Preterm labor without either intra-amniotic inflammation or detectable microbes in the amniotic cavity (control group, n = 88);
2. Preterm labor with intra-amniotic inflammation but without detectable microbes in the amniotic cavity (sterile intra-amniotic inflammation [SIAI] group, n = 19); and
3. Preterm labor with detectable microbes in the amniotic cavity and intra-amniotic inflammation (proven intra-amniotic infection [IAI] group, n = 33).

### Amniotic fluid samples

Amniotic fluid was retrieved either by transabdominal amniocentesis under antiseptic conditions using a 22-gauge needle monitored by ultrasound, or by amniocentesis during cesarean delivery under antiseptic conditions. Amniotic fluid samples were transported in capped, sterile syringes to the clinical laboratory and were cultured for aerobic and anaerobic bacteria as well as for genital mycoplasmas, as previously detailed [35]. At the time of collection, an amniotic fluid white blood cell count [102, 103], a glucose concentration [104] and a Gram stain [105] were performed. Concentrations of IL-6 [92] were determined either at the time of collection or from frozen plasma samples. Amniotic fluid samples used in this study were collected between 17.1 and 36.4 weeks of gestation and were stored at −70°C until analysis.

### Preparation of extracellular vesicle fractions

Amniotic fluid samples were thawed at room temperature and treated with Exoquick-TC^™^ (System Biosciences, SBI, Palo Alto, CA, USA) to sediment the extracellular vesicles, according to the manufacturer’s instructions. The resulting supernatants free of extracellular vesicles, and extracellular vesicle pellets re-suspended in the original starting volume, were collected for subsequent cytokine measurement.

### Cytokine measurement

The concentration of 38 cytokines (see S1 Table) was determined with an in-house multiplexed bead-based assay, using methods similar to those previously described [61]. All antibody pairs and protein standards were purchased from R&D Systems (Minneapolis MN, USA), except those for IFN-β (Abcam, Cambridge, UK). Magnetic beads (Luminex Corporation, Austin, TX, USA) with distinct spectral signatures (regions) were coupled to analyte-specific capture antibodies, according to the manufacturer’s recommendations, and stored at 4°C.

Samples and protein standards for the supernatant fluid and intact vesicles were prepared in assay buffer (1% bovine serum albumin in PBS), and lysed samples and standards were prepared in assay buffer with Triton^™^ X-100 at a final concentration of 0.5%. Samples and standards were combined with analyte-specific capture antibody coupled bead mixtures in 96-well flat bottom plates (Nunc, ThermoFisher Scientific, Waltham, MA, USA) and incubated at 4°C overnight. Plates were washed on a magnetic plate washer (405 TS, Biotek Winooski, VT, USA), followed by incubation with polyclonal biotinylated anti-analyte antibodies for 1 hour at room temperature. Plates were washed and incubated for 30 minutes with 16 μg/ml of streptavidin-phycoerythrin (ThermoFisher Scientific, Waltham, MA, USA) in PBS. Plates were washed and beads were re-suspended in PBS and read on a Luminex 200 analyzer (Luminex Corporation, Austin, TX, USA) with acquisition of 100 beads for each region and analyzed using Bioplex Manager software (Bio-Rad Laboratories, Hercules, CA, USA). Analyte concentrations (pg/ml) were determined using 5P regression algorithms and expressed as the mean pg/ml ± S.E. Concentrations were corrected for dilution by ExoQuick-TC^™^ or Triton^™^ X-100. Extracellular vesicle luminal content was calculated as [analyte content of lysed vesicle] − [analyte content of intact vesicles].

### Statistical analyses

#### Analysis of the demographic data

Continuous variables were compared among multiple groups using the Welch’s one–way analysis of variance (ANOVA) [106] or Kruskal-Wallis test, as appropriate. The Fisher’s exact test was used to compare proportions in the analysis of contingency tables.

#### Differences in protein concentration among groups by amniotic fluid compartment

Protein concentrations were offset by adding 1 unit and then log_2_ transformed to improve normality before analysis [107]. The transformed concentrations were then compared between pairs of the preterm labor groups, using the Wilcoxon rank sum test. Fold change in protein concentrations between groups was determined based on the median log transformed values in each group.

To test for differences in the magnitude of change among the preterm labor groups between different compartments (extracellular vesicle surface, extracellular vesicle internal, and amniotic fluid soluble fraction), a quantile regression model for repeated measures [108] was used for analysis—the transformed protein concentration was treated as the dependent variable, while the preterm labor group, the amniotic fluid compartment and gestational age at amniocentesis were treated as fixed effects. Differences in the effect of infection or inflammation on the cytokine abundance between compartments were tested by allowing for interaction terms in the regression models. The R package, *rqpd*, was used to estimate the model coefficients and their significance.

The Benjamini-Hochberg procedure [109] was employed to correct for multiple comparisons, and a q-value (corrected p-value) of less than 0.05 was considered a statistically significant result.

All analyses were performed using software packages within the R statistical environment [110].

#### Prediction of preterm delivery

An additional analysis was conducted to determine whether amniotic fluid compartmentalized protein abundance measured before 32 weeks of gestation is predictive of the time of delivery (<32 vs ≥32 weeks of gestation). Random forest prediction models [111] were built for each of the three amniotic fluid compartments separately, as well as using data from all three amniotic fluid compartments at the same time. The *randomForest* in R was used to fit the models while allowing for 1,000 decision trees in each random forest model. Leave-one-out cross validation was used to estimate prediction performance indicators, including the area under the receiver operating characteristic (ROC) curves (AUC), sensitivity, and specificity.

## Results

### Clinical characteristics of the study population

Clinical characteristics of the patient population are summarized in Table 1. No significant differences in maternal age, body mass index, gestational age at amniocentesis, gravidity, parity, history of previous preterm delivery, and fetal gender were found among the 3 preterm labor groups. Gestational age at delivery, birthweight, 1- and 5-minute Apgar scores, frequency of clinical chorioamnionitis, amniotic fluid white blood cell count, and amniotic fluid glucose concentration varied significantly among the groups, consistent with clinical presentation. Among the patients with preterm labor with intra-amniotic infection, microorganisms were detected in the amniotic fluid through cultivation in 16 (48.5%) cases and through PCR/ESI-MS in 29 (88%) cases (S2 Table). The most common microorganisms detected included *Sneathia spp*. (n = 6), *Fusobacterium nucleatum* (n = 6), *Ureaplasma parvum* (n = 4), and *Ureaplasma urealyticum* (n = 4).

**Table 1:** Clinical characteristics of the study population.

	PTL without either intra-amniotic inflammation or proven intra-amniotic infection (N = 88)	PTL with sterile intra-amniotic inflammation (N = 19)	PTL with intra-amniotic infection (N = 33)	*P*
**Maternal**				
*Maternal Age (years)*	24.4(5.4)	24.3(4.3)	26.5(6.5)	0.223
BMI*	25.7(7.2)	25.6(5.3)	29(8.2)	0.163
*Gestation Age at Amniocentesis*	26.7(5.3)	29.1(4.9)	25.7(3.8)	0.051
*African American Ethnicity*	77/88(87.5%)	15/19(78.9%)	26/33(78.8%)	0.345
*Smoking*	25/88(28.4%)	3/19(15.8%)	10/33(30.3%)	0.51
Alcohol*	6/86(7%)	1/19(5.3%)	1/33(3%)	0.868
Drugs*	20/87(23%)	8/19(42.1%)	4/32(12.5%)	0.057
**Obstetric History**				
*Primigravida*	22/88(25%)	4/19(21.1%)	4/33(12.1%)	0.307
*Term Deliveries*	39/88(44.3%)	7/19(36.8%)	16/33(48.5%)	0.705
*Preterm Deliveries*	34/88(38.6%)	5/19(26.3%)	7/33(21.2%)	0.171
**Delivery**				
Gestation Age at Delivery (weeks)*	33.7(5.1)	29.5(4.8)	26.3(3.9)	<0.001
Induced Labor*	10/85(11.8%)	1/19(5.3%)	2/32(6.2%)	0.633
C/S*	17/85(20%)	4/19(21.1%)	7/33(21.2%)	1
Vaginal*	66/85(77.6%)	13/19(68.4%)	22/33(66.7%)	0.388
VBAC*	2/85(2.4%)	2/19(10.5%)	4/33(12.1%)	0.052
**Fetal**				
Fetal Male Gender*	46/84(54.8%)	12/19(63.2%)	16/33(48.5%)	0.598
Birth Weight g*	2171.5(888.7)	1512(769.5)	1010.5(602.1)	<0.001
Apgar 1 min < 5*	14/85(16.5%)	6/19(31.6%)	21/33(63.6%)	<0.001
Apgar 5 min < 7*	14/85(16.5%)	7/19(36.8%)	20/33(60.6%)	<0.001
*Amniotic Fluid Glucose (mg/dl) < 14 mg/dl*[104]*	2/83(2.4%)	2/17(11.8%)	16/30(53.3%)	<0.001
*Amniotic Fluid WBC (cells/mm3) ≥ 50 cells/mm3*[102]*	2/79(2.5%)	3/16(18.8%)	17/30(56.7%)	<0.001
Amniotic Fluid RBC (cells/mm3)*	11(3–48.8)	63(10–320)	23(6–120)	0.054ʷ
Amniocentesis to Delivery (days interval)*	43.5(16.2–78.8)	2(1–3.5)	1(0–3)	<0.001ʷ
***Complications***				
*Preterm Premature Rupture of the Membranes*	10/88(11.4%)	2/19(10.5%)	3/33(9.1%)	1
*Small for Gestational Age Neonates*	11/88(12.5%)	1/19(5.3%)	0/33(0%)	0.071
*Preeclampsia*	1/88(1.1%)	0/19(0%)	0/33(0%)	1
*Clinical Chorioamnionitis*	3/88(3.4%)	2/19(10.5%)	8/33(24.2%)	0.002

Continuous variable data are presented as the mean (standard deviation) when the differences among groups were assessed by ANOVA; or as median (interquartile range) when the Kruskal-Wallis test was performed. Count data are presented as number (%) and were compared using Fisher's exact test.

* Contain missing data

^ʷ^ Kruskal-Wallis one-way ANOVA

### Differences in protein concentration by amniotic fluid compartment among the groups

**Fig 1:**
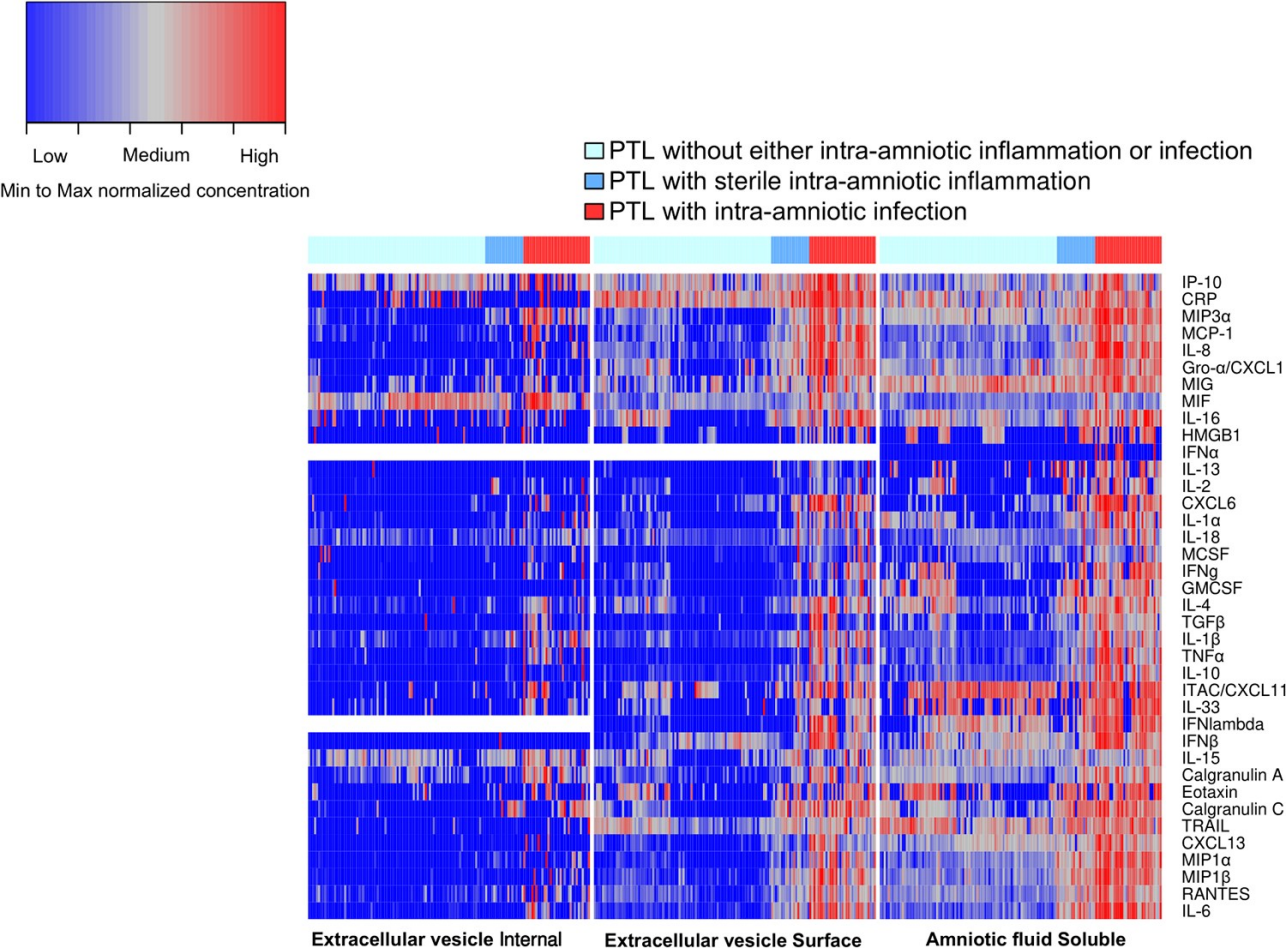
Heatmap of protein concentrations on the extracellular vesicle surface, within the vesicle and in the soluble fraction of amniotic fluid. The protein concentrations were offset by adding 1 unit, log_2_ transformed, and then normalized (min-max normalization) separately for each amniotic fluid compartment.

**Fig 2:**
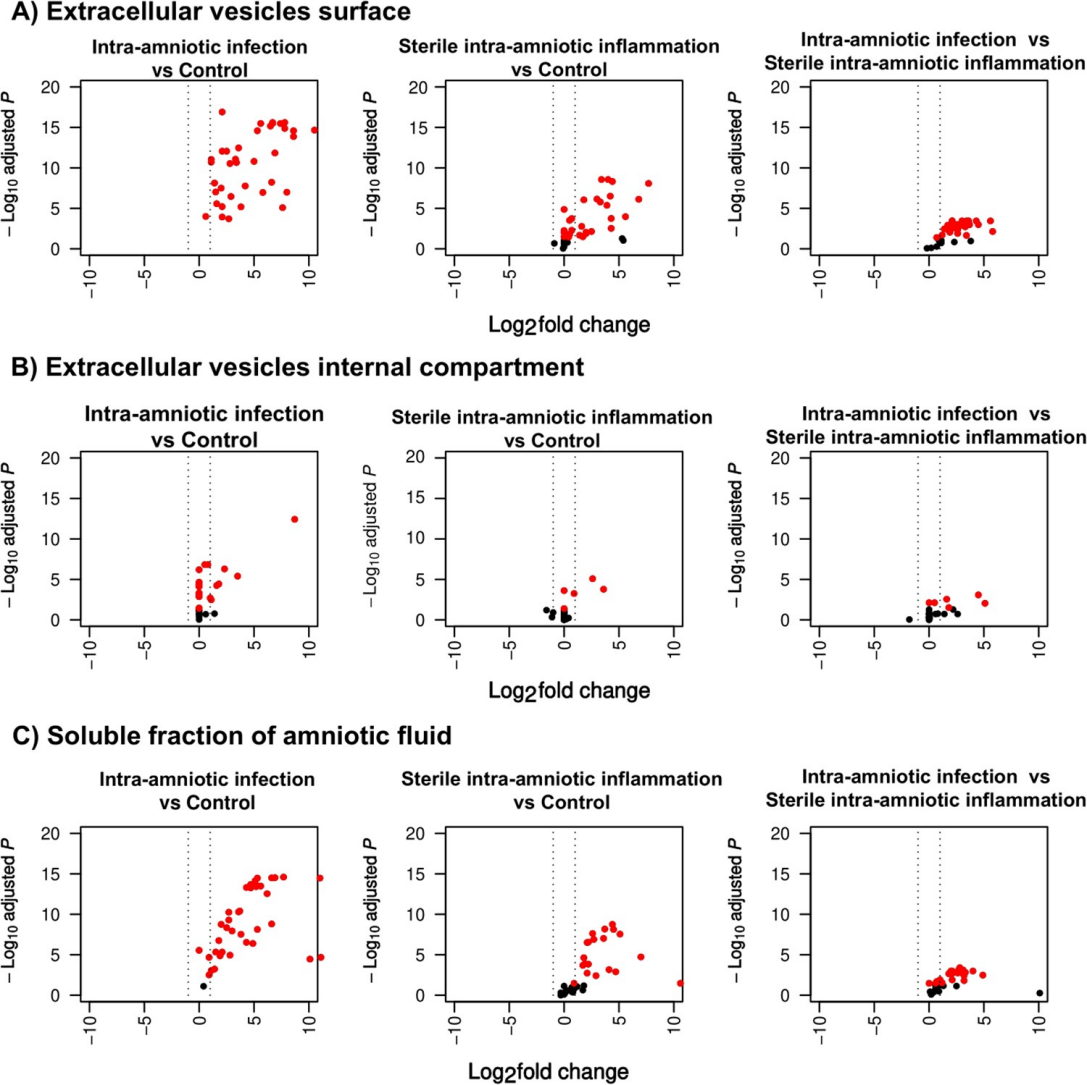
Volcano plot of—log_10_ transformed p-values against log_2_ transformed fold changes illustrating the changes in protein abundance with intra-amniotic infection and with sterile intra-amniotic inflammation. Differentially abundant proteins (q-value < 0.05) are highlighted in red. Changes in protein concentrations were most evident when comparing preterm labor with intra-amniotic infection to preterm labor without either intra-amniotic inflammation or proven intra-amniotic infection on the extracellular vesicle surface or in the soluble fraction of amniotic fluid. Control: preterm labor without either intra-amniotic inflammation or proven intra-amniotic infection.

Protein concentrations by preterm labor group and amniotic fluid compartment are summarized as box plots in S1 Fig and data is available in S3 Table. Intra-amniotic inflammation, regardless of the detection of microorganisms, was associated with a pronounced expression of cytokines on the extracellular vesicle surface and in the soluble fraction of amniotic fluid (Fig 1). Pair-wise comparisons of protein concentration by amniotic fluid compartment are presented in Table 2 and illustrated in volcano plots (Fig 2). For some comparisons, even though the median protein concentrations were identical (log_2_ fold change = 0) in the comparison groups, the Wilcoxon rank sum test returned a significant p-value (e.g. IL-1α: preterm labor with intra-amniotic infection vs preterm labor without either intra-amniotic inflammation or proven intra-amniotic infection in the internal compartment of vesicles Table 2). In most cases, this was due to non-detection of protein (S4 Table) in majority of the samples resulting in median concentrations being zero for the two groups being compared. However, as was the case for IL-1α (S1 Fig), among the samples in which the protein was detected, the concentrations were higher in one group compared to the other. Therefore, these comparisons were retained.

**Table 2:** Compartmentalized differential concentration analysis.

	Extracellular Vesicle Surface			Extracellular Vesicle Internal			Amniotic Fluid Soluble		
	IAI vs Control	SIAI vs Control	IAI vs SIAI	IAI vs Control	SIAI vs Control	IAI vs SIAI	IAI vs Control	SIAI vs Control	IAI vs SIAI
**IL-1α**	3.4***	0**	3.4*	0***	*0*^.^	0	5.3***	2.1**	3.2*
**IL-1β**	3.6***	1.8***	1.9**	2.3***	0.9***	1.4	5.6***	2.7***	2.9***
**IL-2**	1.4***	0	1.4**	0***	0*	0	4.3***	1.8^.^	2.5^.^
**IL-4**	3.3***	1.6**	1.7**	1.8***	0	1.8*	2.5***	2.2***	0.4
**IL-6**	6.7***	3.4***	3.3**	1.6***	0	1.6**	7.7***	4.4***	3.2**
**IL-8**	7.4***	4***	3.4***	0**	0	0	5.3***	2.6***	2.7***
**IL-10**	2.5***	0.7***	1.8**	0***	0	0	4.7***	2.1***	2.6**
**IL-13**	1.1***	0***	1.1				3.8***	2.9**	0.9
**IL-15**	2.7***	2.5**	0.2	1.1**	0.2	0.8	*0*.*4*^.^	-0.2	0.6
**IL-16**	6.6***	5.6***	*1*.*1*^.^	0	0	0	1.8***	*1*.*2*^.^	0.6*
**IL-18**	0.6***	0.7**	-0.2	1**	0.4	0.6	2***	0.1	1.9**
**IL-33**	5.8***	0	5.8**	0***	0	0^.^	2.1***	0	2.2**
**Calgranulin A**	5***	2*	3**	3.5***	-1	4.5***	2.7***	0.9*	1.8**
**Calgranulin C**	8.6***	7.7***	1	0.8***	2.6***	-1.8	5.2***	5.1***	0.1
**Eotaxin-1**	7.6***	*5*.*3*^.^	2.3	0**	0	0	11.1***	10.6*	0.5
**GMCSF**	1.5***	0.4*	1.2*	0	0		4.9***	4.7**	0.2
**Gro-α/CXCL-1**	6.9***	4.3***	2.5**	0^.^	0	0	3.7***	1.7***	2**
**HMGB1**	3.8***	*0*^.^	3.8	0*	0	0	10.1***	*0*^.^	10.1
**IFNγ**	2.8***	0.5*	2.3**	0***		0	6.6***	1.7	4.9**
**IP-10**	4.2***	1.7*	2.6**	1.4	-1.1	2.6	1.5***	0.6	1*
**ITAC/CXCL-11**	8***	5.4^.^	2.6*	0***	0	0	0.9**	-0.3	1.2*
**MCSF**	1.1***	0.5***	0.7*	0**	0	0	1.9***	0.6	*1*.*3*^.^
**MCP-1**	7.8***	4.2***	3.6***	0^.^	0	0	5.1***	1.9***	3.1***
**MIG**	2.9***	2**	*0*.*9*^.^	0^.^	0	0	1.4***	0.8	*0*.*6*^.^
**MIP-1α**	7.8***	3.3***	4.5**	0***	0***	0	6.9***	3.7***	3.2**
**MIP-1β**	6.5***	4.4***	2.1**	0**	0	0	6.6***	4.5***	2.1**
**MIP-3α**	10.5***	6.8***	3.7**	8.7***	3.6***	5.1**	5.2***	2.2***	3**
**RANTES**	5.3***	3***	2.3**	0^.^	0	0	4.3***	3.6***	*0*.*8*^.^
**TGFβ**	2.1***	0	2.1***	0***	0	0**	6.2***	4.1***	2.1***
**TNFα**	2.1***	0**	2.1***	0.5***	0	0.5**	4.7***	1.8***	2.9***
**CRP**	2***	0.3	1.7**	0	0	0	2.8***	0.7	2.1*
**TRAIL**	2.1***	1.4*	0.7	0	0	0	1.1***	0.9	0.2
**CXCL6**	8.6***	4.3**	4.3***	0**	0	0	11***	7***	4***
**CXCL13**	6.7***	3.9***	2.7**	0*		0	2.7***	0.7	2***
**MIF**	1.6***	-0.1	1.7**	0.6	*-1*.*6*^.^	*2*.*2*^.^	0.9***	0.2	0.7*
**IFNα**							0***		0*
**IFNβ**	2.1***	-0.9	3***				3.6***	0.8	2.8***
**IFNλ**	5.6***	0*	5.6***				3***	-0.3	3.3**

Log_2_ fold changes in protein abundance among the three preterm labor groups are presented for each compartment. Statistical significance was assessed by Wilcoxon rank sum test. IAI, preterm labor with intra-amniotic infection; SIAI, preterm labor with sterile intra-amniotic inflammation; Control, preterm labor without either intra-amniotic inflammation or proven intra-amniotic infection

Significance code for p-values:

<0.001 “***”,

<0.01 “**”,

< 0.05 “*”

#### Extracellular vesicle surface

On the surface of extracellular vesicles, median concentrations of 37 proteins were significantly increased (q-value <0.05) in preterm labor with intra-amniotic infection compared to preterm labor without either intra-amniotic inflammation or proven intra-amniotic infection. The highest fold change (FC) was observed for MIP-3α (log_2_ FC = 10.5) followed by CXCl6 (log_2_ FC = 8.6) and Calgranulin C (log_2_ FC = 8.6). Median concentrations of 28 cytokines were significantly higher in preterm labor with sterile intra-amniotic inflammation compared to preterm labor without either intra-amniotic inflammation or proven intra-amniotic infection, with the highest fold changes being observed for Calgranulin C (log2 FC = 7.7), MIP-3α (log2 FC = 6.8), and IL-6 (log2 FC = 5.6). The comparison of intra-amniotic infection to sterile intra-amniotic inflammation groups identified 28 mediators with increased concentration [e.g., IL-33 (log2 FC = 5.8), IFN-λ (log2 FC = 5.6), and MIP-1α (log2 FC = 4.5), among others].

#### Extracellular vesicle internal compartment

In the internal compartment of the extracellular vesicles, the median concentrations of 24 cytokines were significantly higher in preterm labor with intra-amniotic infection compared to preterm labor without either intra-amniotic inflammation or proven intra-amniotic infection. MIP-3α (log_2_ FC = 8.7), Calgranulin A (log_2_ FC = 3.5), and IL-1β (log_2_ FC = 2.3) showed the highest increase in concentration in the presence of intra-amniotic infection.

When comparing preterm labor with sterile intra-amniotic inflammation to preterm labor without either intra-amniotic inflammation or proven intra-amniotic infection, median concentrations of 5 cytokines [e.g. MIP-3α (log2 FC = 3.6), Calgranulin C (log2 FC = 2.6), and IL-1β (log2 FC = 0.9)] were significantly increased. Similarly, six cytokines [e.g., MIP-3α (log2 FC = 5.1), Calgranulin A (log2 FC = 4.5), and IL-4 (log2 FC = 1.8)] showed a significant increase in concentration in preterm labor with intra-amniotic infection compared to preterm labor with sterile intra-amniotic inflammation.

#### Soluble fraction of amniotic fluid

In the soluble fraction of amniotic fluid, median concentrations of 37 cytokines were significantly higher in preterm labor with intra-amniotic infection compared preterm labor without either intra-amniotic inflammation or proven intra-amniotic infection, with eotaxin-1, CXCL-6, and HMGB1 being the most increased (log_2_ FC >10 for all). When comparing preterm labor with sterile intra-amniotic inflammation to preterm labor without either intra-amniotic inflammation or proven intra-amniotic infection, 20 cytokines showed significantly increased abundance, such as eotaxin-1 (log_2_ FC = 10.6), CXCL6 (log_2_ FC = 7), and Calgranulin C (log_2_ FC = 5.1) (Table 2). Finally, 26 cytokines [e.g., IFN-γ (log_2_ FC = 4.9), CXCL6 (log_2_ FC = 4), and IFN-λ (log_2_ FC = 3.3), among others] also showed a significant increase in preterm labor with intra-amniotic infection compared to preterm labor with sterile intra-amniotic inflammation.

### Compartment-dependent differences in protein abundance among three preterm labor groups

**Fig 3:**
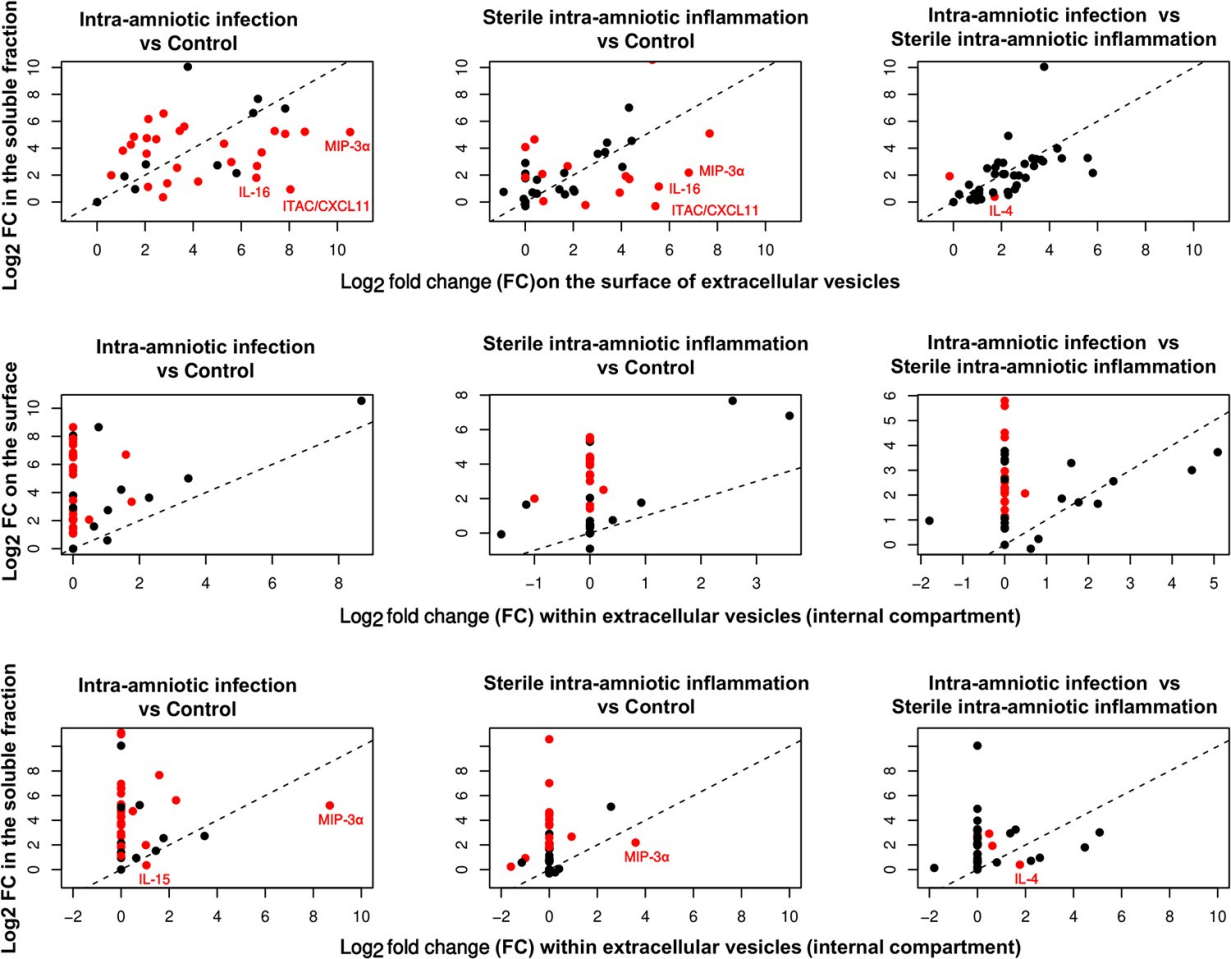
Scatter plot of fold changes in protein abundance with intra- amniotic infection and with sterile intra-amniotic inflammation between pairs of amniotic fluid compartments. Proteins for which the changes were significantly different between compartments are highlighted in red. Data represents log_2_ fold changes between groups. Control: preterm labor without either intra-amniotic inflammation or proven intra-amniotic infection.

Quantile regression models were used to assess if the differences in protein abundance among preterm labor groups were significantly different among the three amniotic fluid compartments. For each pair-wise comparison among the study groups, the differences in log_2_-fold changes between the pairs of amniotic fluid compartments are provided in Table 3. Significant differences highlighted in Fig 3 (scatter plots of paired log_2_ fold changes on the differences observed among the amniotic fluid compartments), are summarized below:

**Table 3:** Amniotic fluid between-compartment differences in the magnitude of changes in cytokine concentrations among preterm labor groups.

	PTL with intra-amniotic infection vs PTL without either intra-amniotic inflammation or proven intra-amniotic infection			PTL with sterile intra-amniotic inflammation vs PTL without either intra-amniotic inflammation or proven intra-amniotic infection			PTL with intra-amniotic infection vs PTL with sterile intra-amniotic inflammation		
	Surface vs Internal	Soluble vs Internal	Soluble vs Surface	Surface vs Internal	Soluble vs Internal	Soluble vs Surface	Surface vs Internal	Soluble vs Internal	Soluble vs Surface
**IL-1α**	3.4**	5.3***	1.9*	0	2.1	2.1	3.4	3.2	-0.2
**IL-1β**	1.3	3.3***	2***	0.9	1.8***	0.9**	0.5	1.5	1
**IL-2**	1.4***	4.3***	2.9***	0	1.8	1.8	1.4***	2.5	1.1
**IL-4**	1.5***	0.7	-0.8*	1.6**	2.2***	0.6	-0.1	-1.4*	-1.3*
**IL-6**	5.1***	6.1***	1	3.4***	4.4***	1	1.7	1.6	-0.1
**IL-8**	7.4***	5.3***	-2.1**	4***	2.6***	-1.4	3.4	2.7	-0.7
**IL-10**	2.5***	4.7***	2.2***	0.7	2.1***	1.4**	1.8*	2.6***	0.8
**IL-13**	1.1**	3.8***	2.7***	0	2.9	2.9	1.1*	0.9	-0.2
**IL-15**	1.6	-0.7*	-2.3***	2.3*	-0.4	-2.7***	-0.6	-0.2	0.4
**IL-16**	6.6***	1.8**	-4.8***	5.6**	1.2	-4.4***	1.1	0.6	-0.5
**IL-18**	-0.4	1**	1.4**	0.3	-0.3	-0.6*	-0.8	1.3*	2.1***
**IL-33**	5.8*	2.1	-3.7	0	0	0	5.8*	2.2	-3.6
**Calgranulin A**	1.5	-0.8	-2.3	3**	1.9*	-1.1	-1.5	-2.7	-1.2
**Calgranulin C**	7.8	4.4	-3.4**	5.1	2.5	-2.6**	2.8	1.9	-0.9
**Eotaxin-1**	7.6*	11.1***	3.5***	5.3	10.6***	5.3*	2.3	0.5	-1.8
**GMCSF**	1.5***	4.9***	3.4***	0.4	4.7***	4.3***	1.2**	0.2	-1
**Gro-α/CXCL-1**	6.9***	3.7***	-3.2**	4.3***	1.7***	-2.6*	2.5**	2	-0.5
**HMGB1**	3.8	10.1	6.3	0	0	0	3.8	10.1	6.3
**IFNγ**	2.8***	6.6***	3.8***	0.5	1.7	1.2	2.3**	4.9	2.6
**IP-10**	2.8	0.1	-2.7***	2.8	1.7	-1.1	0	-1.6	-1.6
**ITAC/CXCL-11**	8	0.9	-7.1**	5.4*	-0.3	-5.7*	2.6	1.2	-1.4
**MCSF**	1.1***	1.9***	0.8	0.5	0.6	0.1	0.7	1.3	0.6
**MCP-1**	7.8**	5.1	-2.7***	4.2***	1.9**	-2.3*	3.6	3.1	-0.5
**MIG**	2.9	1.4	-1.5*	2	0.8	-1.2	0.9	0.6	-0.3
**MIP-1α**	7.8***	6.9***	-0.9	3.3**	3.7***	0.4	4.5*	3.2	-1.3
**MIP-1β**	6.5***	6.6***	0.1	4.4***	4.5***	0.1	2.1*	2.1	0
**MIP-3α**	1.8	-3.5***	-5.3***	3.2	-1.4*	-4.6***	-1.4	-2.1	-0.7
**RANTES**	5.3***	4.3**	-1**	3*	3.6**	0.6	2.3*	0.8	-1.5
**TGFβ**	2.1**	6.2***	4.1***	0	4.1***	4.1***	2.1*	2.1	0
**TNFα**	1.6***	4.2***	2.6***	0	1.8***	1.8***	1.6***	2.4**	0.8
**CRP**	2***	2.8***	0.8	0.3	0.7	0.4	1.7***	2.1**	0.4
**TRAIL**	2.1***	1.1*	-1**	1.4**	0.9	-0.5	0.7	0.2	-0.5
**CXCL6**	8.6***	11***	2.4***	4.3	7***	2.7	4.3*	4	-0.3
**CXCL13**	6.7***	2.7***	-4***	3.9***	0.7	-3.2***	2.7*	2*	-0.7
**MIF**	1	0.3	-0.7	1.5	1.8*	0.3	-0.5	-1.5	-1
**IFNβ**	2.1***	3.6***	1.5*	-0.9	0.8	1.7	3***	2.8***	-0.2
**IFNλ**	5.6***	3***	-2.6***	0	-0.3	-0.3	5.6***	3.3	-2.3

Differences in the magnitude of changes in cytokine concentration with intra-amniotic infection and with sterile intra-amniotic inflammation were assessed between pairs of amniotic fluid compartments. Data represent differences in log_2_ fold changes (Δ log_2_ FC) reported in Table 2. Statistical significance was assessed using quantile regression models. PTL: preterm labor.

Significance code for p-values:

<0.001 “***”,

<0.01 “**”,

< 0.05 “*”

#### The extracellular vesicle surface compartment versus the extracellular internal compartment

The changes in protein abundance with intra-amniotic inflammation, regardless of the detection of microorganisms, were more pronounced on the extracellular vesicle surface compared to the internal compartment (middle panel in Fig 3 and Table 3). For example, when comparing preterm labor with intra-amniotic infection vs preterm labor without either intra-amniotic inflammation or proven intra-amniotic infection, fold changes of 16 proteins were higher when assessed on the extracellular vesicle surface than internal to extracellular vesicles.

#### The extracellular vesicle surface compartment versus the amniotic fluid soluble fraction

A comparison of the data collected on the extracellular vesicle surface and in the soluble fraction of amniotic fluid revealed differences in the fold changes in protein abundance with intra-amniotic infection and with sterile intra-amniotic inflammation as follows: some cytokines showed significantly stronger associations on the extracellular vesicle surface and others in the soluble fraction of amniotic fluid (top panel in Fig 3 and Table 3). For example, when comparing preterm labor with intra-amniotic infection vs preterm labor without either intra-amniotic inflammation or proven intra-amniotic infection, fold changes of 15 proteins were significantly higher when assessed on the surface of extracellular vesicles compared to the soluble amniotic fluid fraction; whereas fold changes of 13 proteins were higher in the soluble fraction of amniotic fluid compared to the extracellular vesicle surface.

#### The extracellular vesicle internal compartment versus the amniotic fluid soluble fraction

The changes in protein abundance with intra-amniotic inflammation, regardless of detected microorganisms, were generally lower when assessed within the extracellular vesicles than in the soluble fraction of amniotic fluid, with a few notable exceptions (bottom panel in Fig 3 and Table 3). For e.g., the fold changes in MIP-3α with intra-amniotic infection and with sterile intra-amniotic inflammation were higher within vesicles compared to the soluble fraction of amniotic fluid (preterm labor with intra-amniotic infection vs preterm labor without either intra-amniotic inflammation or proven intra-amniotic infection: Δ log_2_ FC = 3.5; preterm labor with sterile intra-amniotic inflammation vs preterm labor without either intra-amniotic inflammation or proven intra-amniotic infection: Δ log_2_ FC = 1.4). Similarly, for IL-4, the change in protein abundance with intra-amniotic infection vs sterile intra-amniotic inflammation was higher within extracellular vesicles compared to that in the soluble fraction of amniotic fluid (Δ log_2_ FC = 1.4).

### Prediction of preterm delivery based on compartmentalized proteomic profiles of amniotic fluid

Of the 138 cases for which gestational age at delivery was available, 22 delivered at term (≥ 37 weeks), 61 delivered early preterm (< 32 weeks), and the 55 delivered late preterm (at or after 32 but before 37 weeks).

**Fig 4:**
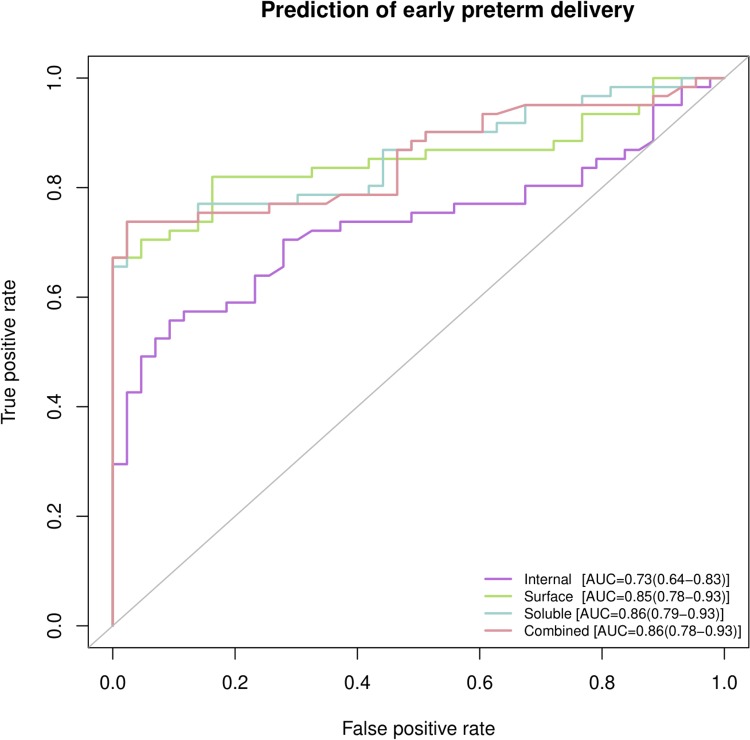
Receiver operating characteristic (ROC) curves of multivariate predictive models for early preterm delivery (<32 weeks of gestation). The Area Under the Receiver Operating Characteristic Curve (AUC) and 95% confidence interval are reported.

For the subset of cases where amniocentesis was performed before 32 weeks of gestation, the predictive value of the compartmentalized cytokine concentrations for early preterm delivery was evaluated by building univariate and multivariate prediction models. The performance of prediction models is summarized in Table 4 that gives the AUC statistic of univariate models by amniotic fluid compartment, in Table 5 that provides the performance metrics (sensitivity, and specificity) of multivariate models, and Fig 4 that displays the Receiver Operating Characteristic curves of multivariate models by amniotic fluid compartment.

**Table 4:** Prediction performance of early preterm delivery by single protein prediction models.

	Extracellular vesicle Surface		Extracellular vesicle Internal		Amniotic fluid Soluble	
	AUC	q-value	AUC	q-value	AUC	q-value
**IL-1α**	0.71(0.64–0.79)	<0.001	0.6(0.54–0.67)	0.050	0.68(0.58–0.78)	0.003
**IL-1β**	0.77(0.68–0.86)	<0.001	0.68(0.58–0.78)	0.012	0.77(0.68–0.86)	<0.001
**IL-2**	0.66(0.58–0.75)	0.002	0.57(0.5–0.64)	0.127	0.65(0.56–0.74)	0.006
**IL-4**	0.73(0.64–0.83)	<0.001	0.61(0.51–0.71)	0.114	0.73(0.63–0.83)	<0.001
**IL-6**	0.87(0.8–0.94)	<0.001	0.59(0.5–0.69)	0.127	0.84(0.76–0.92)	<0.001
**IL-8**	0.88(0.81–0.94)	<0.001	0.57(0.5–0.63)	0.127	0.85(0.77–0.93)	<0.001
**IL-10**	0.76(0.67–0.85)	<0.001	0.53(0.45–0.61)	0.546	0.81(0.73–0.9)	<0.001
**IL-13**	0.67(0.6–0.73)	<0.001	0.51(0.49–0.52)	0.515	0.71(0.62–0.79)	<0.001
**IL-15**	0.62(0.52–0.72)	0.032	0.54(0.43–0.65)	0.546	0.51(0.4–0.62)	0.879
**IL-16**	0.69(0.59–0.79)	0.001	0.56(0.48–0.65)	0.210	0.67(0.57–0.78)	0.004
**IL-18**	0.6(0.49–0.71)	0.098	0.62(0.51–0.72)	0.114	0.68(0.58–0.78)	0.003
**IL-33**	0.65(0.57–0.73)	0.002	0.61(0.54–0.68)	0.050	0.62(0.52–0.73)	0.033
**Calgranulin A**	0.73(0.64–0.82)	<0.001	0.61(0.5–0.71)	0.127	0.74(0.65–0.84)	<0.001
**Calgranulin C**	0.79(0.71–0.86)	<0.001	0.64(0.57–0.71)	0.012	0.85(0.78–0.92)	<0.001
**Eotaxin-1**	0.67(0.58–0.76)	0.002	0.57(0.5–0.64)	0.127	0.63(0.52–0.73)	0.025
**GMCSF**	0.63(0.55–0.71)	0.007	0.47(0.43–0.52)	0.240	0.67(0.57–0.77)	0.004
**Gro-α/CXCL1**	0.73(0.64–0.82)	<0.001	0.57(0.5–0.64)	0.146	0.77(0.67–0.86)	<0.001
**HMGB1**	0.6(0.52–0.68)	0.026	0.53(0.48–0.57)	0.453	0.61(0.52–0.7)	0.036
**IFNγ**	0.69(0.61–0.78)	<0.001	0.59(0.53–0.64)	0.050	0.69(0.59–0.78)	0.001
**IP10**	0.7(0.6–0.8)	0.001	0.57(0.46–0.68)	0.339	0.68(0.57–0.78)	0.004
**ITAC/CXCL11**	0.64(0.54–0.74)	0.014	0.58(0.51–0.66)	0.114	0.56(0.45–0.67)	0.303
**MCSF**	0.76(0.67–0.84)	<0.001	0.56(0.49–0.63)	0.172	0.66(0.55–0.76)	0.009
**MCP1**	0.83(0.75–0.91)	<0.001	0.48(0.38–0.58)	0.711	0.85(0.78–0.93)	<0.001
**MIG**	0.68(0.58–0.78)	0.002	0.53(0.43–0.63)	0.636	0.59(0.48–0.7)	0.130
**MIP-1α**	0.82(0.75–0.89)	<0.001	0.58(0.51–0.65)	0.114	0.87(0.8–0.94)	<0.001
**MIP-1β**	0.84(0.77–0.92)	<0.001	0.57(0.5–0.63)	0.127	0.83(0.75–0.92)	<0.001
**MIP-3α**	0.78(0.7–0.87)	<0.001	0.75(0.67–0.83)	<0.001	0.87(0.8–0.94)	<0.001
**RANTES**	0.78(0.7–0.87)	<0.001	0.61(0.51–0.7)	0.114	0.81(0.73–0.9)	<0.001
**TGFβ**	0.71(0.63–0.79)	<0.001	0.58(0.52–0.65)	0.114	0.76(0.67–0.84)	<0.001
**TNFα**	0.76(0.69–0.82)	<0.001	0.67(0.61–0.74)	0.001	0.81(0.73–0.89)	<0.001
**CRP**	0.65(0.55–0.76)	0.010	0.51(0.42–0.59)	0.896	0.64(0.53–0.75)	0.020
**TRAIL**	0.59(0.48–0.7)	0.115	0.52(0.46–0.58)	0.595	0.57(0.45–0.68)	0.265
**CXCL6**	0.77(0.69–0.85)	<0.001	0.57(0.51–0.63)	0.114	0.83(0.76–0.91)	<0.001
**CXCL13**	0.79(0.71–0.87)	<0.001	0.54(0.49–0.58)	0.202	0.74(0.64–0.83)	<0.001
**MIF**	0.59(0.48–0.7)	0.122	0.54(0.42–0.65)	0.595	0.66(0.56–0.77)	0.006
**IFNα**					0.57(0.52–0.61)	0.018
**IFNβ**	0.61(0.5–0.71)	0.074			0.76(0.67–0.85)	<0.001
**IFNλ**	0.74(0.67–0.81)	<0.001			0.7(0.6–0.8)	0.001

Area Under the Receiver Operating Characteristic Curve (AUC) for univariate prediction models using data collected from each amniotic fluid compartment separately, are presented along with their 95% confidence intervals and q-values (adjusted p-values from Wilcoxon rank sum test for differences between early preterm delivery and moderate to late preterm or term delivery).

**Table 5:** Prediction performance of early preterm delivery by multivariate protein prediction models.

Model	Sensitivity	Specificity	Positive likelihood ratio	Negative likelihood ratio	AUC
**Amniotic fluid Soluble**	0.74 (0.61–0.84)	0.93 (0.81–0.99)	10.57 (3.51–31.82)	0.28 (0.18–0.43)	0.86 (0.79–0.93)
**Extracellular vesicle Surface**	0.7 (0.57–0.81)	0.91 (0.78–0.97)	7.58 (2.94–19.54)	0.33 (0.22–0.49)	0.85 (0.78–0.93)
**Extracellular vesicle Internal**	0.67 (0.54–0.79)	0.72 (0.56–0.85)	2.41 (1.44–4.02)	0.45 (0.3–0.68)	0.73 (0.64–0.83)
**Combined**	0.74 (0.61–0.84)	0.93 (0.81–0.99)	10.57 (3.51–31.82)	0.28 (0.18–0.43)	0.86 (0.78–0.93)

Prediction performance metrics for early preterm delivery (<32 weeks of gestation) is given for multivariate predictive models, using as predictors the compartmentalized protein concentration profiles. Statistics are presented along with their 95% confidence intervals. The prevalence of early preterm delivery(<32 weeks of gestation) in the United States was 1.59% in 2017 [112].

For 33 cytokines, the AUC estimates obtained from univariate models based on their extracellular vesicle surface concentrations were significantly higher than 0.5 [e.g. IL-8 0.88(0.81–0.94), IL-6 0.87(0.8–0.94), MIP-1β 0.84(0.77–0.92)]. Moreover, the AUC statistic for 16 of these cytokines (e.g. IL-15, MCSF, MIG, and ITAC/CXCL11, among others) was higher based on data collected on the extracellular vesicle surface than the data collected in the soluble fraction of amniotic fluid, yet the difference failed to reach statistical significance. The AUC estimates based on their extracellular vesicle internal concentrations were generally lower than the extracellular vesicle surface or amniotic fluid soluble counterparts (Table 4).

The performance indicators of multivariate models based on cytokine measurements in the extracellular vesicle internal, extracellular vesicle surface, and amniotic fluid soluble compartments were overall similar [EV Internal AUC = 0.73 (0.64–0.83), EV Surface AUC = 0.85 (0.78–0.93), Soluble fraction AUC = 0.86 (0.79–0.93)]. The top five ranked cytokines, based on their importance in prediction, were found in these compartments: EV surface: IL-6, IL-8, MCP-1, MIP-1β, and MIP-1α; EV internal: Calgranulin C, IP-10, MIP-3α, IL-1β, and MIF; and amniotic fluid soluble: MIP-1α, MIP-1β, MIP-3α, IL-6, and IL-8. There was no significant improvement in the cross-validated point estimate of AUC of a multi-variate early preterm delivery prediction model when information from all three compartments was used [AUC = 0.86 (0.78–0.93)] as compared to individual compartments.

## Discussion

Pathologic insults to the feto-placental unit are often clinically expressed as complications of pregnancy, such as preterm labor with intact membranes and preterm prelabor rupture of the membranes [113–115]. A contemporary paradigm of the initiation of labor and delivery, both at term and preterm, involves the activation of intrauterine inflammatory signaling pathways and a shift to a more pro-inflammatory intra-amniotic environment [35, 60, 61, 96–101, 103, 116–128].

In addition to these canonical signaling pathways, recent studies identified the release of extracellular vesicles as a significant pathway for intercellular communication [129, 130]. The release of extracellular vesicles is an evolutionarily conserved effector and intercellular-signaling pathway and has been described in archeacian [131], prokaryotic [132], and eukaryotic [133–137] cells. Extracellular vesicles, including exosomes (nanovesicles of late endoplasmic reticulum origin and released via the fusion of multivesicular bodies with the plasma membrane) and ectosomes (nanovesicles formed by the direct budding of the plasma membrane) [130, 138], are derived from distinct biogenic pathways. They may share common functional activities but are profoundly different in their origin, biogenesis, distribution, release mechanisms, and response to stimuli [139]. In contrast to well-characterized endocrine signaling pathways, extracellular vesicle-dependent communication allows the exchange of proteins [140], RNA [140, 141], and DNA [142], with conformational fidelity.

Extracellular vesicles regulate inflammatory and immune processes [89, 143–158] and are present in amniotic fluid [75, 76, 81, 159, 160]. Amnion-derived extracellular vesicles are biologically active as mediators of proliferation, apoptosis, immune responses, angiogenesis, and inflammation [78, 79, 81] and may participate in labor-associated paracrine signaling [86, 90, 161]. Nanovesicles may affect these changes either directly, via the pro- and anti- inflammatory proteins carried on their surface and within their lumen, or indirectly, via the induction of inflammatory pathways in the target cells with which they engage [86].

To gain insight into the contribution of proteins packaged in extracellular vesicles, we established the profile and compartmentalization of extracellular vesicle-associated proteins in amniotic fluid obtained from women with preterm labor. We evaluated the distribution of 38 cytokines between the vesicle surface and intra-vesicle compartments. Their concentration in extracellular vesicle-free amniotic fluid was also determined for each sample. These measurements allowed us to test whether there was an advantage to measuring cytokine concentrations in the extracellular vesicle compartments, as opposed to unfractionated amniotic fluid, in predicting preterm delivery. We have specifically addressed the following research questions:

### Does the cytokine abundance internal to and on the extracellular vesicle surface change with sterile intra-amniotic inflammation and with intra-amniotic infection in women with preterm labor?

We found that intra-amniotic inflammation, regardless of detected microbial invasion of the amniotic fluid cavity, was associated with an increased abundance of cytokines internal to and on the surface of extracellular vesicles. Earlier we reported on the general increase of cytokines [61] in unfractionated amniotic fluid obtained from preterm labor cases with intra-amniotic infection and sterile intra-amniotic inflammation. These results are reconfirmed herein, but here, for the first time we report on changes in cytokines quantified internally and on the surface of extracellular vesicles in this subset of women with preterm labor. Development of the pro-inflammatory vesicle surface was most evident in intra-amniotic infection, for which the surface expression of 37 cytokines was increased. Of these, 36 cytokines were also increased in the soluble fraction of amniotic fluid, and 24 had increased concentrations within the extracellular vesicles.

The extracellular vesicle associated proteins showing the strongest increase in concentration with intra-amniotic infection were MIP-3α, Calgranulin C, CXCL6, ITAC/CXCL11, MCP1, MIP-1α, Eotaxin 1, IL-8, Gro-α/CXCL1, and IL-6 on the surface, and MIP-3α and Calgranulin A in the lumen. The up-regulation of these pro-inflammatory cytokines (IL-6), chemokines (MIP-3α, CXCL6, ITAC/CXCL11, MCP1, MIP-1α, Eotaxin 1, IL-8, Gro-α/CXCL1) and antimicrobial polypeptides (Calgranulin C) results from the activation of the innate immune system which includes the recognition of pathogen-associated molecular patterns (PAMPs) by pattern recognition receptors (e.g. Toll-like receptors, C-reactive protein) [61, 113, 162–167]. This is plausible given the observed increase in the concentration of the C-reactive protein (CRP) in the soluble fraction of the amniotic fluid in preterm labor with intra-amniotic infection.

The top ranked proteins based on the magnitude of change in their concentrations with sterile intra-amniotic inflammation were Calgranulin C, MIP-3α, IL-16, MIP-1b, Groα/CXCL1, CXCL6, MCP-1, IL-8, CXCL13, and IL-6 on the surface of extracellular vesicles and MIP-3α, and Calgranulin C in the lumen of extracellular vesicles. The overexpression of these inflammatory mediators in the absence of microbes is initiated upon recognition of damage-associated molecular patterns (DAMPs or alarmins) by pattern recognition receptors (e.g. Toll-like receptors, NLRs, etc.) [61, 165, 168–171]. In this study, we observed that concentrations of alarmins, IL-1α [172], and S100 family proteins (Calgranulin A, and Calgranulin C) [173] were significantly increased in the soluble fraction of the amniotic fluid in preterm labor cases with sterile intra-amniotic inflammation. These findings are in line with previous studies showing that the intra-amniotic administration of alarmins induces sterile intra-amniotic inflammation and preterm birth in mice [174, 175]. The mechanisms whereby DAMPs induce sterile intra-amniotic inflammation and preterm birth involve the activation of the NLRP3 inflammasome [99, 124, 175–179]

The association of intra-amniotic inflammation with or without detected microbes with preterm parturition syndrome and the role of cytokines in these processes are well documented [60, 61, 93, 96, 97, 180–182]. However, this study shows that there is up-regulation in inflammatory mediators secreted not only as free molecules in amniotic fluid but also as part of the cargo carried by extracellular vesicles. The observed expression and the change in abundance of not only free (soluble) cytokines but also extracellular vesicle associated cytokines with intra-amniotic inflammation/infection may represent urgency and redundancy in the feto-placental unit’s response to environmental stimuli.

While cytokine changes with inflammation, regardless of detected microbial invasion of the amniotic fluid cavity, on the surface of extracellular vesicles correlated with those determined in the soluble fraction of amniotic fluid, the magnitude of the increase was significantly different between these compartments, suggesting that they include different information about the amniotic fluid milieu. Overall, although protein changes in extracellular vesicle surface and the soluble fraction in intra-amniotic inflammation, regardless of detected micro-organisms, were higher than those within the extracellular vesicles, the magnitude of changes within the extracellular vesicles was higher compared to the soluble fraction of amniotic fluid for MIP-3α.

Cytokines contained within the internal compartment are not accessible to routine immunoassay quantification; therefore, this contribution has not been incorporated in contemporary models of inflammation-associated parturition. While the relative amount of internal mediators is low, their biological effect may be significant as vesicles can deliver these mediators to the very vicinity of the target cells. The roles of these extracellular vesicle -associated mediators in parturition remain to be established. Previously, we and other investigators have established that encapsulation within extracellular vesicles protects against degradation and affords alternative pathways for engagement with target cells and transport pathways [183].

### Is compartmentalized amniotic fluid protein concentration profiling useful in the development of biomarkers for early preterm delivery?

To determine the potential of amniotic fluid compartmentalized protein profiling for developing new biomarkers of preterm labor and other obstetrical complications, we have developed univariate and multi-variate prediction models for early preterm delivery (gestational age ≤32 weeks). For all but one cytokine (TRAIL), the AUC estimates obtained from either the extracellular vesicle surface or the amniotic fluid soluble fraction concentrations were significantly above 0.5. The AUC statistic for univariate models based on data collected on the extracellular vesicle surface for IL-6, IL-8, MCP-1, MIP-1α, and MIP-1β was greater than 0.8. This is consistent with our previous report of the association between unfractionated amniotic fluid concentrations of these proteins and early preterm delivery in patients diagnosed with a short cervix [62]. Although prediction performances (AUC) for individual cytokines were different between compartments, the overall performance of the multivariate models was statistically similar among the different amniotic fluid compartments. This finding can be explained in part by the redundancy of the cytokine network, but it does not preclude that compartmentalized profiling could improve prediction performance for other phenotypes.

### Strengths and limitations

The main strength of this study is its novelty component, which is the compartmentalized profiling of proteins in the amniotic fluid for three phenotypes of preterm labor. Another strength is the moderate sample size that enabled not only detection of differences among groups within a given compartment, but also detection of significant differences in the magnitude of changes between groups across the different compartments. The use of cross-validation to avoid overfitting in multi-variate models was also a strength of the analysis. Limitations are related to the variability in the gestational ages at sampling of amniotic fluid, and the lack of amniotic fluid samples collected during second or early third trimester from mothers without preterm labor.

## Conclusions

The data obtained in this study are consistent with our hypothesis that amniotic fluid proteins are differentially expressed and grouped within the extracellular vesicle and soluble amniotic fluid fraction, and compartment-specific profiles characterize clinical subgroups of preterm labor. Intra-amniotic inflammation with and without detectable microorganisms was associated with the differential packaging of extracellular vesicles cytokines. Proteins packaged within the extracellular vesicles contribute to total amniotic fluid concentrations. Although the current study points to possible improvement in biomarker prediction based on compartmentalized profiling, further de-convolution of the heterogeneity of preterm labor and delivery is warranted.

## Supporting information

S1 Fig Box plots of transformed protein concentrations by preterm labor group and amniotic fluid compartment. Protein concentrations were offset by adding 1 unit and then log_2_ transformed before plotting. Control: preterm labor without either intra-amniotic inflammation or proven intra-amniotic infection.
(TIFF)

S1 Table Protein annotation. Full names of the 38 proteins measured on the surface and within extracellular vesicles and in the soluble fraction of amniotic fluid are listed along with official symbols (www.uniprot.org) of the genes coding for these proteins.
(DOCX)

S2 Table Microorganisms detected in the amniotic fluid. Each line corresponds to one of the 33 patients with intra amniotic infection.
(DOCX)

S3 Table Proteomic dataset. Concentrations (pg/ml) of 38 proteins in amniotic fluid compartments (internal, surface, and soluble) for each sample (row) are provided. ID: anonymized identifier indicator of the pregnant mother, GAatAmnio: gestational age at the time of amniocentesis (weeks); GAatDelivery: gestational age at the time of delivery (weeks); Group: preterm labor subgroup where IAI refers to intra-amniotic infection and SIAI refers to sterile intra-amniotic inflammation.
(XLSX)

S4 Table Detection of proteins. The number (and the proportion) of cases where a non-zero protein concentration was detected is presented by preterm labor group and by amniotic fluid compartment. PTL: preterm labor, EV: extracellular vesicle, AF: amniotic fluid.
(DOCX)
