# Supplementary figures and images for: Compartmentalized profiling of amniotic fluid cytokines in women with preterm labor
Source: PLoS One. 2020 Jan 16;15(1):e0227881. doi: 10.1371/journal.pone.0227881 (PMC6964819; doi:10.1371/journal.pone.0227881)

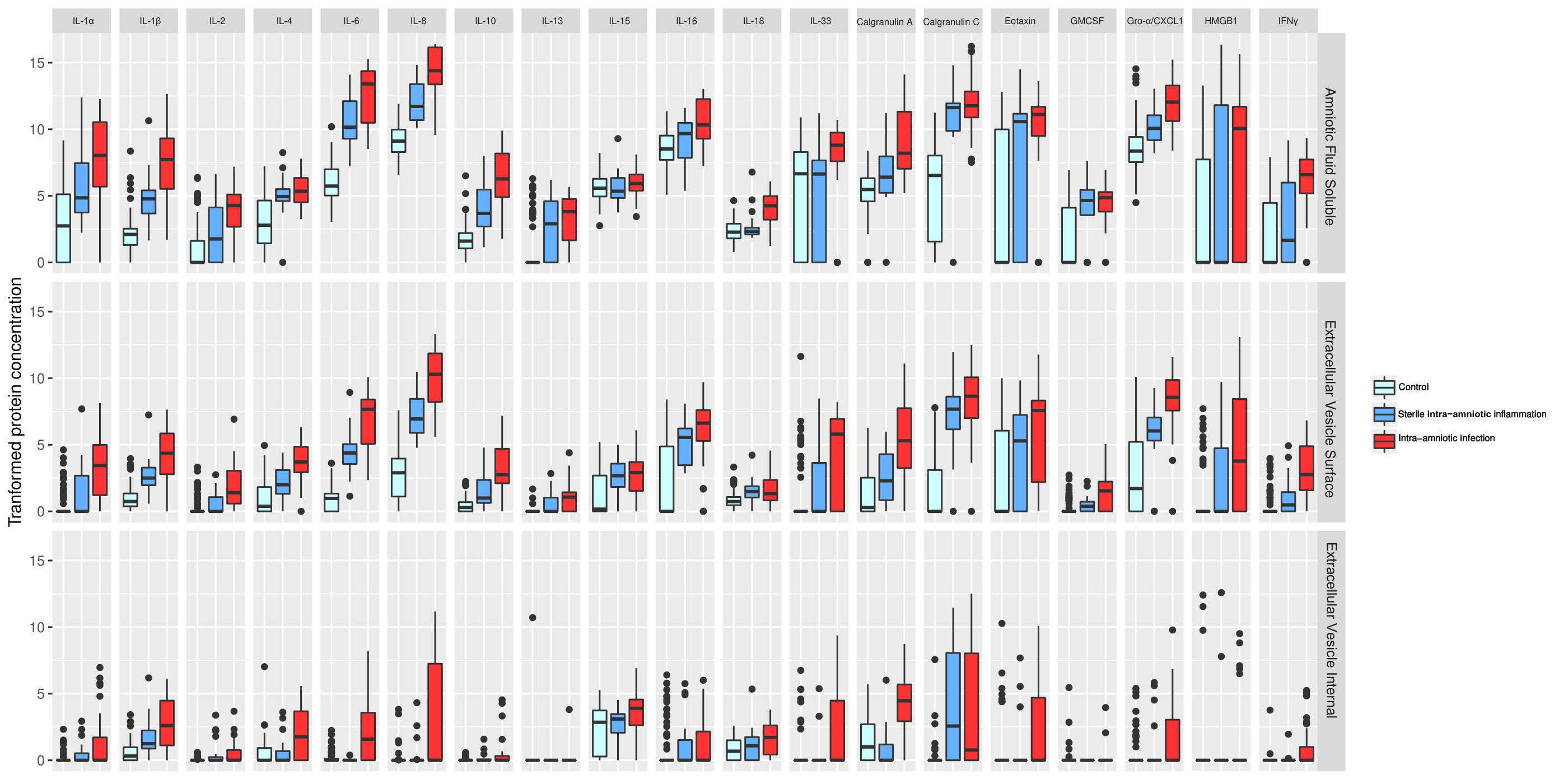

Supplement: S1 Fig — Protein concentrations were offset by adding 1 unit and then log2 transformed before plotting. Control: preterm labor without either intra-amniotic inflammation or proven intra-amniotic infection. (TIFF) [file pone.0227881.s001.tiff]
