# Supplementary material for: Compartmentalized profiling of amniotic fluid cytokines in women with preterm labor
Source: PLoS One. 2020 Jan 16;15(1):e0227881. doi: 10.1371/journal.pone.0227881 (PMC6964819; doi:10.1371/journal.pone.0227881)
Supplement: S1 Table — Full names of the 38 proteins measured on the surface and within extracellular vesicles and in the soluble fraction of amniotic fluid are listed along with official symbols (www.uniprot.org) of the genes coding for these proteins. (DOCX) [file pone.0227881.s002.docx]

**S1Table. Protein Annotation.**

|  | **Genes** | **Protein Names** |
| --- | --- | --- |
| **CRP** | CRP, PTX1 | C-reactive protein [Cleaved into: C-reactive protein(1-205)] |
| **CXCL13** | CXCL13, BCA1, BLC, SCYB13 | C-X-C motif chemokine 13 (Angie) (B cell-attracting chemokine 1) (BCA-1) (B lymphocyte chemoattractant) (CXC chemokine BLC) (Small-inducible cytokine B13) |
| **CXCL6** | CXCL6, GCP2, SCYB6 | C-X-C motif chemokine 6 (Chemokine alpha 3) (CKA-3) (Granulocyte chemotactic protein 2) (GCP-2) (Small-inducible cytokine B6) [Cleaved into: Small-inducible cytokine B6, N-processed variant 1; Small-inducible cytokine B6, N-processed variant 2; Small-inducible cytokine B6, N-processed variant 3] |
| **HMGB1** | HMGB1, HMG1 | High mobility group protein B1 (High mobility group protein 1) (HMG-1) |
| **IFNγ** | IFNG | Interferon gamma (IFN-gamma) (Immune interferon) |
| **IL-10** | IL10 | Interleukin-10 (IL-10) (Cytokine synthesis inhibitory factor) (CSIF) |
| **IL-13** | IL13, NC30 | Interleukin-13 (IL-13) |
| **IL-15** | IL15 | Interleukin-15 (IL-15) |
| **IL-16** | IL16 | Pro-interleukin-16 [Cleaved into: Interleukin-16 (IL-16) (Lymphocyte chemoattractant factor) (LCF)] |
| **IL-18** | IL18, IGIF, IL1F4 | Interleukin-18 (IL-18) (Iboctadekin) (Interferon gamma-inducing factor) (IFN-gamma-inducing factor) (Interleukin-1 gamma) (IL-1 gamma) |
| **IL-1α** | IL1A, IL1F1 | Interleukin-1 alpha (IL-1 alpha) (Hematopoietin-1) |
| **IL-1β** | IL1B, IL1F2 | Interleukin-1 beta (IL-1 beta) (Catabolin) |
| **IL-2** | IL2 | Interleukin-2 (IL-2) (T-cell growth factor) (TCGF) (Aldesleukin) |
| **IL-33** | IL33, C9orf26, IL1F11, NFHEV | Interleukin-33 (IL-33) (Interleukin-1 family member 11) (IL-1F11) (Nuclear factor from high endothelial venules) (NF-HEV) [Cleaved into: Interleukin-33 (95-270); Interleukin-33 (99-270); Interleukin-33 (109-270)] |
| **IL-4** | IL4 | Interleukin-4 (IL-4) (B-cell stimulatory factor 1) (BSF-1) (Binetrakin) (Lymphocyte stimulatory factor 1) (Pitrakinra) |
| **IL-6** | IL6, IFNB2 | Interleukin-6 (IL-6) (B-cell stimulatory factor 2) (BSF-2) (CTL differentiation factor) (CDF) (Hybridoma growth factor) (Interferon beta-2) (IFN-beta-2) |
| **MIF** | MIF, GLIF, MMIF | Macrophage migration inhibitory factor (MIF) (EC 5.3.2.1) (Glycosylation-inhibiting factor) (GIF) (L-dopachrome isomerase) (L-dopachrome tautomerase) (EC 5.3.3.12) (Phenylpyruvate tautomerase) |
| **IFNλ** | IFNL1, IL29, ZCYTO21 | Interferon lambda-1 (IFN-lambda-1) (Cytokine Zcyto21) (Interleukin-29) (IL-29) |
| **TRAIL** | TNFSF10, APO2L, TRAIL | Tumor necrosis factor ligand superfamily member 10 (Apo-2 ligand) (Apo-2L) (TNF-related apoptosis-inducing ligand) (Protein TRAIL) (CD antigen CD253) |
| **ITAC/CXCL11** | CXCL11, ITAC, SCYB11, SCYB9B | C-X-C motif chemokine 11 (Beta-R1) (H174) (Interferon gamma-inducible protein 9) (IP-9) (Interferon-inducible T-cell alpha chemoattractant) (I-TAC) (Small-inducible cytokine B11) |
| **GMCSF** | CSF2, GMCSF | Granulocyte-macrophage colony-stimulating factor (GM-CSF) (Colony-stimulating factor) (CSF) (Molgramostin) (Sargramostim) |
| **MIP1α** | CCL3, G0S19-1, MIP1A, SCYA3 | C-C motif chemokine 3 (G0/G1 switch regulatory protein 19-1) (Macrophage inflammatory protein 1-alpha) (MIP-1-alpha) (PAT 464.1) (SIS-beta) (Small-inducible cytokine A3) (Tonsillar lymphocyte LD78 alpha protein) [Cleaved into: MIP-1-alpha(4-69) (LD78-alpha(4-69))] |
| **MIP1β** | CCL4, LAG1, MIP1B, SCYA4 | C-C motif chemokine 4 (G-26 T-lymphocyte-secreted protein) (HC21) (Lymphocyte activation gene 1 protein) (LAG-1) (MIP-1-beta(1-69)) (Macrophage inflammatory protein 1-beta) (MIP-1-beta) (PAT 744) (Protein H400) (SIS-gamma) (Small-inducible cytokine A4) (T-cell activation protein 2) (ACT-2) [Cleaved into: MIP-1-beta(3-69)] |
| **TGFβ** | TGFB1, TGFB | Transforming growth factor beta-1 proprotein [Cleaved into: Latency-associated peptide (LAP); Transforming growth factor beta-1 (TGF-beta-1)] |
| **MIP3α** | CCL20, LARC, MIP3A, SCYA20 | C-C motif chemokine 20 (Beta-chemokine exodus-1) (CC chemokine LARC) (Liver and activation-regulated chemokine) (Macrophage inflammatory protein 3 alpha) (MIP-3-alpha) (Small-inducible cytokine A20) [Cleaved into: CCL20(1-67); CCL20(1-64); CCL20(2-70)] |
| **CALGRANULIN C** | S100A12 | Protein S100-A12 (CGRP) (Calcium-binding protein in amniotic fluid 1) (CAAF1) (Calgranulin-C) (CAGC) (Extracellular newly identified RAGE-binding protein) (EN-RAGE) (Migration inhibitory factor-related protein 6) (MRP-6) (p6) (Neutrophil S100 protein) (S100 calcium-binding protein A12) [Cleaved into: Calcitermin] |
| **MCSF** | CSF1 | Macrophage colony-stimulating factor 1 (CSF-1) (M-CSF) (MCSF) (Lanimostim) [Cleaved into: Processed macrophage colony-stimulating factor 1] |
| **EOTAXIN** | CCL11, SCYA11 | Eotaxin (C-C motif chemokine 11) (Eosinophil chemotactic protein) (Small-inducible cytokine A11) |
| **GROα/CXCL1** | CXCL1, GRO, GRO1, GROA, MGSA, SCYB1 | Growth-regulated alpha protein (C-X-C motif chemokine 1) (GRO-alpha(1-73)) (Melanoma growth stimulatory activity) (MGSA) (Neutrophil-activating protein 3) (NAP-3) [Cleaved into: GRO-alpha(4-73); GRO-alpha(5-73); GRO-alpha(6-73)] |
| **RANTES** | CCL5, D17S136E, SCYA5 | C-C motif chemokine 5 (EoCP) (Eosinophil chemotactic cytokine) (SIS-delta) (Small-inducible cytokine A5) (T cell-specific protein P228) (TCP228) (T-cell-specific protein RANTES) [Cleaved into: RANTES(3-68); RANTES(4-68)] |
| **CALGRANULIN A** | S100A8, CAGA, CFAG, MRP8 | Protein S100-A8 (Calgranulin-A) (Calprotectin L1L subunit) (Cystic fibrosis antigen) (CFAG) (Leukocyte L1 complex light chain) (Migration inhibitory factor-related protein 8) (MRP-8) (p8) (S100 calcium-binding protein A8) (Urinary stone protein band A) |
| **IL-8** | CXCL8, IL8 | Interleukin-8 (IL-8) (C-X-C motif chemokine 8) (Chemokine (C-X-C motif) ligand 8) (Emoctakin) (Granulocyte chemotactic protein 1) (GCP-1) (Monocyte-derived neutrophil chemotactic factor) (MDNCF) (Monocyte-derived neutrophil-activating peptide) (MONAP) (Neutrophil-activating protein 1) (NAP-1) (Protein 3-10C) (T-cell chemotactic factor) [Cleaved into: MDNCF-a (GCP/IL-8 protein IV) (IL8/NAP1 form I); Interleukin-8 ((Ala-IL-8)77) (GCP/IL-8 protein II) (IL-8(1-77)) (IL8/NAP1 form II) (MDNCF-b); IL-8(5-77); IL-8(6-77) ((Ser-IL-8)72) (GCP/IL-8 protein I) (IL8/NAP1 form III) (Lymphocyte-derived neutrophil-activating factor) (LYNAP) (MDNCF-c) (Neutrophil-activating factor) (NAF); IL-8(7-77) (GCP/IL-8 protein V) (IL8/NAP1 form IV); IL-8(8-77) (GCP/IL-8 protein VI) (IL8/NAP1 form V); IL-8(9-77) (GCP/IL-8 protein III) (IL8/NAP1 form VI)] |
| **IFNα** | IFNA1, IFNA13 | Interferon alpha-1/13 (IFN-alpha-1/13) (Interferon alpha-D) (LeIF D) |
| **IFNβ** | IFNB1, IFB, IFNB | Interferon beta (IFN-beta) (Fibroblast interferon) |
| **IP-10** | CXCL10, INP10, SCYB10 | C-X-C motif chemokine 10 (10 kDa interferon gamma-induced protein) (Gamma-IP10) (IP-10) (Small-inducible cytokine B10) [Cleaved into: CXCL10(1-73)] |
| **MCP1** | CCL2, MCP1, SCYA2 | C-C motif chemokine 2 (HC11) (Monocyte chemoattractant protein 1) (Monocyte chemotactic and activating factor) (MCAF) (Monocyte chemotactic protein 1) (MCP-1) (Monocyte secretory protein JE) (Small-inducible cytokine A2) |
| **MIG** | CXCL9, CMK, MIG, SCYB9 | C-X-C motif chemokine 9 (Gamma-interferon-induced monokine) (Monokine induced by interferon-gamma) (HuMIG) (MIG) (Small-inducible cytokine B9) |
| **TNFα** | TNF, TNFA, TNFSF2 | Tumor necrosis factor (Cachectin) (TNF-alpha) (Tumor necrosis factor ligand superfamily member 2) (TNF-a) [Cleaved into: Tumor necrosis factor, membrane form (N-terminal fragment) (NTF); Intracellular domain 1 (ICD1); Intracellular domain 2 (ICD2); C-domain 1; C-domain 2; Tumor necrosis factor, soluble form] |
