# Supplementary material for: Compartmentalized profiling of amniotic fluid cytokines in women with preterm labor
Source: PLoS One. 2020 Jan 16;15(1):e0227881. doi: 10.1371/journal.pone.0227881 (PMC6964819; doi:10.1371/journal.pone.0227881)
Supplement: S2 Table — Each line corresponds to one of the 33 patients with intra amniotic infection. (DOCX) [file pone.0227881.s003.docx]

**S2Table. Microorganisms detected in the amniotic fluid**.

|  | **Microorganisms determined by cultivation** | **Microorganisms determined by**  **polymerase chain reaction/electrospray ionization-mass spectrometry** |
| --- | --- | --- |
| 1 | *Gardnerella vaginalis* | *Sneathia spp.* |
| 2 | *Peptostreptococcus spp., Cytomegalovirus* | *Parvovirus B19,*  *Cytomegalovirus* |
| 3 | *Ureaplasma urealyticum* | *Ureaplasma parvum, Fusobacterium nucleatum* |
| 4 | *Streptococcus agalactiae* | *Streptococcus agalactiae* |
| 5 | *Candida spp.* | *Propionibacterium acnes , Fusobacterium nucleatum, Candida albicans* |
| 6 | *Fusobacterium spp.* | *Fusobacterium nucleatum* |
| 7 | *Bacteroides spp., Mobiluncus spp.,Clostridium sporogenes* | *Fusobacterium nucleatum* |
| 8 | *Bacteroides ureolyticus* | *Streptobacillus moniliformis, Sneathia* |
| 9 | *Coagulase-negative staphylococci* |  |
| 10 | *Staphylococcus aureus* | *Staphylococcus aureus* |
| 11 | *Peptostreptococcus spp., Streptococcus anginosus, Propionibacterium acnes* |  |
| 12 | *Candida spp.* | *Candida albicans* |
| 13 | *Bacteroides ureolyticus, Lachnoanaerobaculum spp.* |  |
| 14 | *Ureaplasma urealyticum* |  |
| 15 | *Gram-negative bacilli spp.* | *Sneathia spp.* |
| 16 | *Gardnerella vaginalis, Coccobacillus spp.* | *Sneathia spp.* |
| 17 |  | *Streptococcus agalactiae, Parvovirus B19, Human herpesvirus 2* |
| 18 |  | *Acinetobacter junii* |
| 19 |  | *Lactobacillus kefiranofaciens* |
| 20 |  | *Sneathia* |
| 21 |  | *Sneathia, Fusobacterium nucleatum, Bacteroides fragilis, Bacteroides thetaiotaomicron* |
| 22 |  | *Streptococcus spp. , Pseudomonas mendocina* |
| 23 |  | *Propionibacterium acnes , Acinetobacter junii , Cytomegalovirus* |
| 24 |  | *Cytomegalovirus* |
| 25 |  | *Ureaplasma urealyticum* |
| 26 |  | *Ureaplasma urealyticum* |
| 27 |  | *Cytomegalovirus* |
| 28 |  | *Human herpesvirus 1* |
| 29 |  | *Propionibacterium acnes* |
| 30 |  | *Fusobacterium nucleatum* |
| 31 |  | *Ureaplasma parvum* |
| 32 |  | *Ureaplasma parvum* |
| 33 |  | *Ureaplasma parvum* |
