# Supplementary material for: Compartmentalized profiling of amniotic fluid cytokines in women with preterm labor
Source: PLoS One. 2020 Jan 16;15(1):e0227881. doi: 10.1371/journal.pone.0227881 (PMC6964819; doi:10.1371/journal.pone.0227881)
Supplement: S4 Table — The number (and the proportion) of cases where a non-zero protein concentration was detected is presented by preterm labor group and by amniotic fluid compartment. PTL: preterm labor, EV: extracellular vesicle, AF: amniotic fluid. (DOCX) [file pone.0227881.s005.docx]

**S4 Table. Detection of proteins.**

|  | **PTL without either**  **intra-amniotic inflammation**  **or detectable infection**  **(N=88)** | | | **PTL with Sterile**  **intra-amniotic inflammation (N=19)** | | | **PTL with Intra-amniotic**  **infection (N=33)** | | |
| --- | --- | --- | --- | --- | --- | --- | --- | --- | --- |
| **Cytokine** | **EV Internal** | **EV Surface** | **AF Soluble** | **EV Internal** | **EV Surface** | **AF Soluble** | **EV Internal** | **EV Surface** | **AF Soluble** |
| **Calgranulin A** | 52 (59.1%) | 44 (50%) | 84 (95.5%) | 6 (31.6%) | 15 (78.9%) | 18 (94.7%) | 27 (81.8%) | 31 (93.9%) | 33 (100%) |
| **Calgranulin C** | 7 (8%) | 32 (36.4%) | 66 (75%) | 10 (52.6%) | 18 (94.7%) | 18 (94.7%) | 17 (51.5%) | 32 (97%) | 33 (100%) |
| **CRP** | 24 (27.3%) | 88 (100%) | 88 (100%) | 1 (5.3%) | 19 (100%) | 19 (100%) | 4 (12.1%) | 33 (100%) | 33 (100%) |
| **CXCL13** | 2 (2.3%) | 30 (34.1%) | 85 (96.6%) | 1 (5.3%) | 16 (84.2%) | 18 (94.7%) | 4 (12.1%) | 33 (100%) | 33 (100%) |
| **CXCL6** | 6 (6.8%) | 21 (23.9%) | 24 (27.3%) | 1 (5.3%) | 10 (52.6%) | 13 (68.4%) | 8 (24.2%) | 29 (87.9%) | 30 (90.9%) |
| **Eotaxin** | 8 (9.1%) | 30 (34.1%) | 39 (44.3%) | 3 (15.8%) | 11 (57.9%) | 13 (68.4%) | 11 (33.3%) | 25 (75.8%) | 27 (81.8%) |
| **GMCSF** | 6 (6.8%) | 20 (22.7%) | 38 (43.2%) | 0 (0%) | 10 (52.6%) | 15 (78.9%) | 2 (6.1%) | 22 (66.7%) | 31 (93.9%) |
| **Groα/CXCL1** | 13 (14.8%) | 45 (51.1%) | 88 (100%) | 4 (21.1%) | 17 (89.5%) | 19 (100%) | 9 (27.3%) | 32 (97%) | 33 (100%) |
| **HMGB1** | 3 (3.4%) | 14 (15.9%) | 23 (26.1%) | 2 (10.5%) | 6 (31.6%) | 8 (42.1%) | 5 (15.2%) | 17 (51.5%) | 20 (60.6%) |
| **IFNα** | 0 (0%) | 0 (0%) | 0 (0%) | 0 (0%) | 0 (0%) | 0 (0%) | 0 (0%) | 0 (0%) | 8 (24.2%) |
| **IFNβ** | 0 (0%) | 70 (79.5%) | 74 (84.1%) | 1 (5.3%) | 16 (84.2%) | 17 (89.5%) | 0 (0%) | 31 (93.9%) | 33 (100%) |
| **IFNγ** | 2 (2.3%) | 20 (22.7%) | 32 (36.4%) | 2 (10.5%) | 10 (52.6%) | 10 (52.6%) | 10 (30.3%) | 27 (81.8%) | 30 (90.9%) |
| **IFNλ** | 0 (0%) | 11 (12.5%) | 65 (73.9%) | 0 (0%) | 6 (31.6%) | 13 (68.4%) | 0 (0%) | 28 (84.8%) | 29 (87.9%) |
| **IL-10** | 8 (9.1%) | 51 (58%) | 73 (83%) | 5 (26.3%) | 16 (84.2%) | 19 (100%) | 12 (36.4%) | 30 (90.9%) | 33 (100%) |
| **IL-13** | 1 (1.1%) | 3 (3.4%) | 16 (18.2%) | 0 (0%) | 7 (36.8%) | 10 (52.6%) | 1 (3%) | 19 (57.6%) | 25 (75.8%) |
| **IL-15** | 66 (75%) | 44 (50%) | 88 (100%) | 17 (89.5%) | 16 (84.2%) | 19 (100%) | 29 (87.9%) | 27 (81.8%) | 33 (100%) |
| **IL-16** | 20 (22.7%) | 42 (47.7%) | 88 (100%) | 9 (47.4%) | 19 (100%) | 19 (100%) | 10 (30.3%) | 31 (93.9%) | 33 (100%) |
| **IL-18** | 61 (69.3%) | 80 (90.9%) | 88 (100%) | 13 (68.4%) | 18 (94.7%) | 19 (100%) | 28 (84.8%) | 32 (97%) | 33 (100%) |
| **IL-1α** | 9 (10.2%) | 19 (21.6%) | 62 (70.5%) | 6 (31.6%) | 9 (47.4%) | 19 (100%) | 14 (42.4%) | 27 (81.8%) | 31 (93.9%) |
| **IL-1β** | 55 (62.5%) | 70 (79.5%) | 86 (97.7%) | 18 (94.7%) | 19 (100%) | 19 (100%) | 28 (84.8%) | 32 (97%) | 33 (100%) |
| **IL-2** | 6 (6.8%) | 21 (23.9%) | 25 (28.4%) | 5 (26.3%) | 8 (42.1%) | 10 (52.6%) | 10 (30.3%) | 25 (75.8%) | 25 (75.8%) |
| **IL-33** | 10 (11.4%) | 19 (21.6%) | 48 (54.5%) | 2 (10.5%) | 7 (36.8%) | 11 (57.9%) | 15 (45.5%) | 22 (66.7%) | 29 (87.9%) |
| **IL-4** | 37 (42%) | 45 (51.1%) | 67 (76.1%) | 8 (42.1%) | 17 (89.5%) | 18 (94.7%) | 24 (72.7%) | 32 (97%) | 33 (100%) |
| **IL-6** | 26 (29.5%) | 63 (71.6%) | 88 (100%) | 1 (5.3%) | 19 (100%) | 19 (100%) | 19 (57.6%) | 33 (100%) | 33 (100%) |
| **IL-8** | 11 (12.5%) | 81 (92%) | 88 (100%) | 4 (21.1%) | 19 (100%) | 19 (100%) | 12 (36.4%) | 33 (100%) | 33 (100%) |
| **IP-10** | 73 (83%) | 81 (92%) | 88 (100%) | 12 (63.2%) | 19 (100%) | 19 (100%) | 22 (66.7%) | 33 (100%) | 33 (100%) |
| **ITAC/CXCL11** | 8 (9.1%) | 39 (44.3%) | 71 (80.7%) | 3 (15.8%) | 12 (63.2%) | 12 (63.2%) | 14 (42.4%) | 26 (78.8%) | 29 (87.9%) |
| **MCP-1** | 28 (31.8%) | 50 (56.8%) | 88 (100%) | 5 (26.3%) | 19 (100%) | 19 (100%) | 13 (39.4%) | 33 (100%) | 33 (100%) |
| **MCSF** | 9 (10.2%) | 17 (19.3%) | 58 (65.9%) | 3 (15.8%) | 12 (63.2%) | 15 (78.9%) | 12 (36.4%) | 27 (81.8%) | 30 (90.9%) |
| **MIF** | 86 (97.7%) | 88 (100%) | 88 (100%) | 16 (84.2%) | 19 (100%) | 19 (100%) | 29 (87.9%) | 33 (100%) | 33 (100%) |
| **MIG** | 31 (35.2%) | 59 (67%) | 87 (98.9%) | 7 (36.8%) | 17 (89.5%) | 19 (100%) | 16 (48.5%) | 31 (93.9%) | 33 (100%) |
| **MIP-1α** | 6 (6.8%) | 28 (31.8%) | 83 (94.3%) | 8 (42.1%) | 15 (78.9%) | 19 (100%) | 12 (36.4%) | 32 (97%) | 33 (100%) |
| **MIP-1β** | 13 (14.8%) | 54 (61.4%) | 85 (96.6%) | 2 (10.5%) | 19 (100%) | 19 (100%) | 11 (33.3%) | 33 (100%) | 33 (100%) |
| **MIP-3α** | 18 (20.5%) | 42 (47.7%) | 83 (94.3%) | 12 (63.2%) | 18 (94.7%) | 19 (100%) | 28 (84.8%) | 33 (100%) | 33 (100%) |
| **RANTES** | 34 (38.6%) | 61 (69.3%) | 88 (100%) | 9 (47.4%) | 18 (94.7%) | 19 (100%) | 15 (45.5%) | 33 (100%) | 33 (100%) |
| **TGFβ** | 5 (5.7%) | 19 (21.6%) | 36 (40.9%) | 0 (0%) | 5 (26.3%) | 17 (89.5%) | 15 (45.5%) | 28 (84.8%) | 33 (100%) |
| **TNFα** | 8 (9.1%) | 7 (8%) | 57 (64.8%) | 2 (10.5%) | 6 (31.6%) | 18 (94.7%) | 18 (54.5%) | 29 (87.9%) | 32 (97%) |
| **TRAIL** | 7 (8%) | 79 (89.8%) | 86 (97.7%) | 1 (5.3%) | 18 (94.7%) | 19 (100%) | 5 (15.2%) | 32 (97%) | 33 (100%) |
